# Research status of elderly-care robots and safe human-robot interaction methods

**DOI:** 10.3389/fnins.2023.1291682

**Published:** 2023-11-30

**Authors:** Donghui Zhao, Xingwang Sun, Bo Shan, Zihao Yang, Junyou Yang, Houde Liu, Yinlai Jiang, Yokoi Hiroshi

**Affiliations:** ^1^School of Electrical Engineering, Shenyang University of Technology, Shenyang, China; ^2^Tsinghua Shenzhen International Graduate School, Tsinghua University, Shenzhen, China; ^3^Department of Mechanical Engineering and Intelligent Systems, University of Electro-Communications, Tokyo, Japan

**Keywords:** elderly-care robots, nursing mode, safe interaction methods, practicality, acceptability

## Abstract

Faced with the increasingly severe global aging population with fewer children, the research, development, and application of elderly-care robots are expected to provide some technical means to solve the problems of elderly care, disability and semi-disability nursing, and rehabilitation. Elderly-care robots involve biomechanics, computer science, automatic control, ethics, and other fields of knowledge, which is one of the most challenging and most concerned research fields of robotics. Unlike other robots, elderly-care robots work for the frail elderly. There is information exchange and energy exchange between people and robots, and the safe human-robot interaction methods are the research core and key technology. The states of the art of elderly-care robots and their various nursing modes and safe interaction methods are introduced and discussed in this paper. To conclude, considering the disparity between current elderly care robots and their anticipated objectives, we offer a comprehensive overview of the critical technologies and research trends that impact and enhance the feasibility and acceptance of elderly care robots. These areas encompass the collaborative assistance of diverse assistive robots, the establishment of a novel smart home care model for elderly individuals using sensor networks, the optimization of robot design for improved flexibility, and the enhancement of robot acceptability.

## 1 Introduction

According to the latest data from the United Nations, the global population is expected to reach 8 billion by 15 November 2022. The global population will likely grow to around 8.5 billion by 2030 and 9.7 billion by 2050. Concurrently, the elderly population is expected to rise from 771 million in 2022 to 994 million in 2030 and 1.6 billion in 2050. The proportion of individuals aged 65 and over is projected to increase from 9.7% in 2022 to 11.7% in 2030 and further to 16.4% in 2050 (United Nations Department for Economic and Social Affairs, 2023). Moreover, the number of frail elderly individuals unable to engage in physical activity is anticipated to surpass 440 million by 2021 (World Health Organization, 2022). This global aging phenomenon and a low birth rate are increasing the demand for elderly care services. However, statistics from 2019 indicate that the number of nursing staff dedicated to elderly care accounted for only 9% of the professional nursing staff, totaling approximately 15 million individuals worldwide (United Nations, 2019). This glaring disparity between the demand for nursing care and the available resources has become a pressing global issue (Wu et al., 2021). Consequently, the World Health Organization (WHO) and all United Nations Member States approved the “United Nations Decade of Healthy Aging” project in 2020, which emphasizes the provision and accessibility of long-term care for older persons as one of its key areas of focus for the next decade (Arias-Casais et al., 2022). To address these challenges, leveraging robotic nursing technology to effectively and safely attend to the daily needs of the frail elderly population represents a promising solution. This approach helps to bridge the gap between demand and resources and enhances the well-being of elderly individuals with compromised functional abilities.

The field of elderly care has witnessed the emergence of elderly-care robots, which can effectively assist and replace manual caregiving in meeting the daily life needs of elderly individuals. These robots have the potential to alleviate the burden on families and society in providing elderly care, as well as address the critical shortage of nursing staff (Vercelli et al., 2018). Furthermore, they offer promising prospects for improving the quality of life for individuals with compromised functional abilities and contribute to social stability and development (Pilotto et al., 2018). In contrast to traditional service robots (Gonzalez-Aguirre et al., 2021; Lee, 2021) and auxiliary robots (Alboul et al., 2023) that provide a wide range of services for different types of humans or families in various industries, elderly-care robots are tailored to meet the unique needs and challenges of the elderly. However, frail elderly individuals often experience motor function issues such as osteoporosis and muscle weakness. However, frail elderly often experience motor function issues such as osteoporosis and muscle weakness. Additionally, the decline in language, limb, and emotional expression further complicates the accurate conveyance of nursing intentions from elderly individuals with weak functional abilities. This presents significant challenges for delivering appropriate nursing to this particular group, necessitating a high level of expertise in nursing practices. The shortage of nursing staff, coupled with the demanding nature of nursing requirements, has given rise to the development of the robotic nursing industry. The growing demand for high-performance elderly-care robots has provided unprecedented opportunities and challenges.

## 2 Elderly-care robots and their various nursing modes

The elderly-care robot integrates advanced robotics technology with the specific needs of elderly care, enabling accurate, safe, and intelligent nursing to enhance the well-being and level of care for frail elderly. Over the past decade, many countries have implemented significant policies to promote the development of the elderly-care robot industry. Over the past decade, numerous countries have implemented tremendous policies to encourage the development of the elderly-care robot industry. For instance, the National Science Foundation (NSF) funded $37 million in 2016 to support fundamental research on companion robots and home care robots (Bekey and Yuh, 2008). The European Union’s civil robot research and development program, SPARC, allocated 700 million euros from 2014 to 2020 to develop key technologies for robots to address the challenges of an aging society (Huet and Mastroddi, 2016). In 2016, Japan introduced the “Five-Year Plan for Care Robots” into the national “2016 Revitalization Strategy,” investing 100 billion yen to support research into robotic technologies for elderly care. This research covered various aspects, including transfers, toileting, monitoring, bathing, communication, and rehabilitation (Neumann, 2016). The development of high-performance elderly-care robots holds significant importance in light of the global elderly-care landscape. (1) Medical assistant function: Designed to assist with medical tasks with almost no social characteristics, such as human physiological index monitoring and auxiliary analysis. (2) Daily living assistance and rehabilitation training function: Aims to assist frail elderly with simple daily living and care tasks, including feeding, walking, mobility, turning over, postural support, toileting, sleep care, dressing and undressing, and some rehabilitation training tasks. (3) Life companion and emotional interaction function: Enhances the quality of life of elderly individuals through voice communication and companionship. In practical elderly care settings, the integration of these three functions aims to achieve the following objectives:

- Enhancing the autonomy and behavioral abilities of elderly individuals, enabling them to live in a comfortable environment while preserving their dignity.
- Ensuring the safety and companionship of elderly individuals, maintaining their health and mobility, and promoting a healthy lifestyle.
- Effectively distributing caregiving tasks among nursing staff, families, and healthcare organizations, thus optimizing resource allocation and efficiency.

Based on the medical assistant function, daily living assistance and rehabilitation training function, as well as life companion and emotional interaction function of elderly-care robots, and the varying emphases of these functions, we categorize elderly-care robots into three main types: intelligent robots based on medical functionality and physiological index monitoring, life-assisted nursing robots, and companion robots based on emotional interaction and behavior monitoring. The purpose of this classification is to better meet the diverse needs of the elderly and the complexity of real-life elderly care scenarios.

### 2.1 Intelligent robots based on medical functionality and physiological index monitoring

Addressing specific physiological, motor, and cognitive impairments in some elderly individuals, the system aims to complement and enhance medical services based on physiological monitoring and analysis while respecting user privacy. This strengthens the autonomy of elderly individuals and improves their physical and cognitive conditions (Heerink et al., 2006; Stefanie et al., 2017; Etemad-Sajadi and Gomes Dos Santos, 2019).

**Figure 1:**
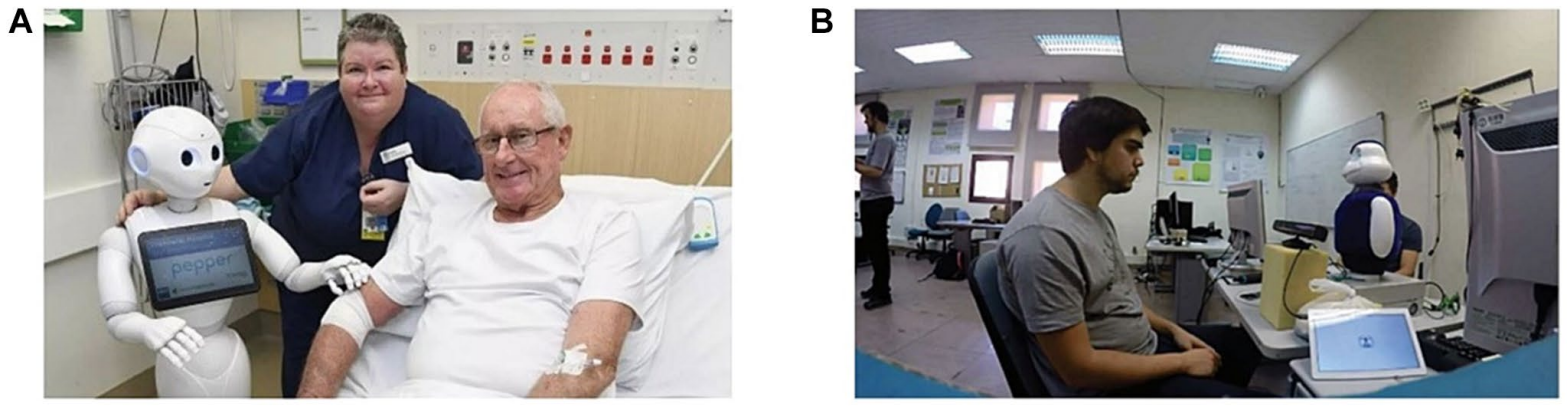
Intelligent robots based on medical functionality and physiological index monitoring. **(A)** PHAROS. **(B)** Mini.

Personal AAL (Personal Ambient Assisted Living; Corcella et al., 2019) and Emerald (Rincon et al., 2019) are examples of systems that remotely measure physiological indicators using high-precision wearable sensors. These systems automatically generate visual health recommendations while assessing the users’ health status. Additionally, the system can provide early intervention for elderly individuals living in the home environment, effectively assess and minimize the long-term impact of various factors on health, and encourage elderly individuals to maintain an active and healthy lifestyle (Alsulami et al., 2017, 2022; Almalki et al., 2022). PHAROS (Physical Assistant Robot System) combines the Pepper robot (Figure 1A) and a motion expert system to analyze users’ motion states during daily training using deep learning methods (Costa et al., 2018; Martinez-Martin et al., 2019). It provides real-time posture correction and positive feedback to encourage and enhance users’ initiative. The system offers personalized training sets and programs, compares the degree of decline based on certified clinical guidelines, and notifies healthcare professionals of potential physical issues. IMBTMMS (Intelligent Mobile Body Temperature Monitoring and Management System; Cui et al., 2021) is designed for elderly care settings. It regularly processes body temperature, heart rate, and blood oxygen data, providing real-time and continuous monitoring, prediction, alerting, and management services. The system’s implementation in nursing homes has achieved large-scale index monitoring and data management, significantly alleviating staff shortages during epidemics and reducing the risk of cross-infection. In summary, Personal AAL and Emerald utilize wearable sensors to assess the health status and physical activity of the elderly, while PHAROS employs visual input to analyze motion states. Proper sensor placement is crucial for accurate measurement results, and ensuring correct order becomes challenging when frail elderly individuals are responsible for sensor installation. Furthermore, these technologies primarily support and assist medical staff rather than directly replacing them. Therefore, medical staff need access to comprehensive physical activity data and daily assessment reports to utilize these systems effectively.

Castillo et al. proposed a robot (Figure 1B) for guiding the rehabilitation of language apraxia (Castillo et al., 2018). The model parameters are trained using oral motion information from standardized elderly individuals. Subsequently, the system captures oral motion and utilizes deep learning methods to evaluate key points, ensuring the correct execution of the treatment regimen. With the assistance of professionals, the system improves muscle movement planning, sequencing, and coordination to produce speech ultimately. Regarding cognitive aspects, CoME effectively analyzes the development.

process of mild cognitive impairment by detecting early signs of abnormal user behavior (COME, 2021). It offers cognitive games, monitors health status, provides recommendations for maintaining a healthy lifestyle, and offers guidance on daily activities, chores, and relevant knowledge for the elderly. MyMemory is a mobile augmented memory system that combines schedules and games to help individuals with traumatic brain injury recover from autobiographical memory deficits (Chang et al., 2018). Clinical research indicates that the system aids in recovering autobiographical memory deficits among elderly individuals. In the systems mentioned above, nursing staff receive personalized reports regarding platform usage, the health status of elderly individuals, and their overall progress. Moreover, professional nursing staff can create tutorials to guide relatives and non-professional caregivers in completing assistance tasks. These systems significantly contribute to treating specific conditions such as mild cognitive impairment, traumatic brain injury, or language apraxia. However, the technologies employed in these systems require the supervision of medical professionals. They are limited to use during formal treatment periods, hindering elderly individuals from independently pursuing physical health and improvement at home. To enhance the versatility of such systems, StayFitLonger (Perez-Marcos et al., 2018) proposes a solution that operates without the supervision of medical professionals. This integrated system combines physical and cognitive training for elderly individuals, offering gamified activities and personalized exercises through mobile devices. It allows users to engage in more extended periods of activity. Additionally, virtual reality technology provides assistance and instructions during exercises, encouraging active participation among elderly individuals. The platform aims to empower elderly individuals to use it autonomously at home and provides various exercise modes based on their flexibility and injury levels.

### 2.2 Life-assisted nursing robots

The care of frail elderly individuals demands a high degree of specialization and remains primarily reliant on manual caregiving. This approach not only entails significant caregiving complexity and intensity but also places substantial reliance on the service proficiency of caregivers. With the development of assistive robots, rehabilitation robots, and human-robot interaction technologies, life-assisting nursing robots and their key technologies are emerging. These robots must adapt to users’ physiological differences and living conditions, enabling them to safely operate in a user-centric environment and provide secure assistance to frail elderly individuals in mobility, household tasks, rehabilitation training, and various daily life demands. In general, these robots can be categorized into three main types: robots for autonomous assistive tasks, rehabilitation robots in elderly care scenarios, and multifunctional nursing robots based on human-robot interaction.

#### 2.2.1 Robots for autonomous assistive tasks

Robots for autonomous assistive tasks can autonomously complete repetitive service or nursing tasks without human intervention. They can help elderly individuals deliver medicines and items, automatically clean excrement, dehumidify, purify, and other functions (Christoforou et al., 2020).

**Figure 2:**
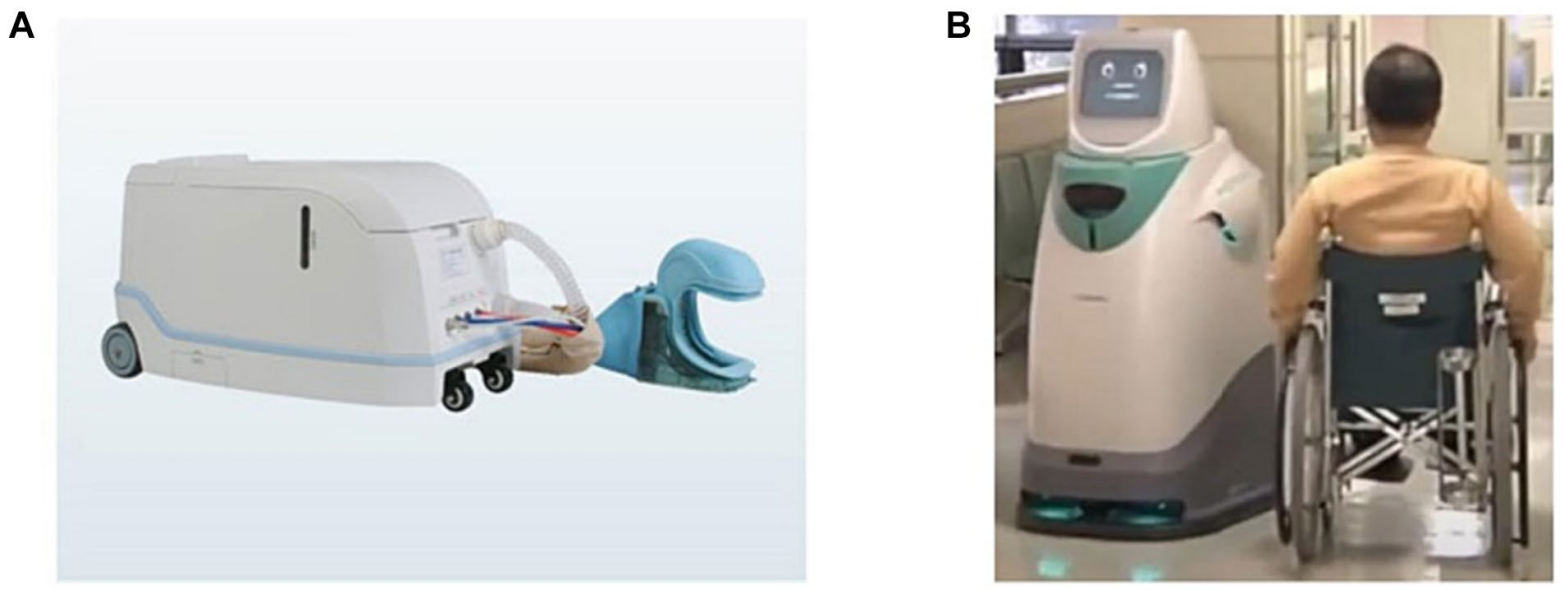
Robots for autonomous assistive tasks. **(A)** XFCS-A. **(B)** HOSPI-R.

The XFCS-A (Qian et al., 2010) nursing robot (Figure 2A) is primarily employed to assist elderly individuals or long-term bedridden patients in defecation. At the same time, cleaning steps such as warm water washing, warm air drying, and negative ion purification are performed on the user’s buttocks. This robot has gained significant popularity in hospitals and homes due to its convenience, safety, and reliability. In 2005, Toshiba Corporation of Japan developed the HOSPI-R series of home care robots (Panasonic’s Autonomous Delivery Robot - HOSPI(R), 2023; Figure 2B), which includes functions such as drug delivery and risk status identification. These robots comprehend user instructions through voice and visual systems, autonomously follow the user, and assess potential risk states in the surrounding environment, providing timely prompts to the operator. To address concerns related to drug or liquid overflow caused by robot acceleration or deceleration, various storage methods for liquids and solids, as well as buffer devices, have been devised to enhance the safety of the drug transportation process (Mertz, 2012). Yanshan University has invented a bidirectional transfer nursing robot. This innovation enables the transfer of frail elderly from hospital beds to stretcher workshops, effectively reducing the labor intensity of nursing staff and avoiding the secondary damage to the user caused by the traditional transfer method (Wang et al., 2016a,b). Additionally, Shenyang University of Technology has developed an intelligent toilet robot with autonomous movement and slip risk identification, as well as an intelligent wheelchair robot capable of adjusting the user’s seating posture (Yu, 2019). To enhance the safety and flexibility of these robots’ usage for elderly individuals with weakened functions, a highly robust follow-up control method has been proposed for the intelligent toilet robot. This involves designing seating trajectories and postures based on the principles of user motion engineering, calculating the control rate of the intelligent toilet robot accordingly, and providing optimal seating postures for users with varying levels of mobility.

#### 2.2.2 Rehabilitation robots in elderly care scenarios

Rehabilitation robots in elderly care scenarios are crucial in assisting frail elderly with rehabilitation training functions in hospitals, nursing homes, homes, and other scenarios.

In the late 20th century, developed countries began to focus on the development of nursing bed robots, which are mainly used for various operations such as rehabilitation training, turning, and defecation of long-term bedridden elderly (Chen, 2016). These nursing bed robots provide upper and lower limb rehabilitation training and muscle massage functions, promoting bone rehabilitation and improving limb blood circulation for individuals whose limbs cannot stretch due to prolonged bedridden conditions. The nursing bed robots also offer vital signs monitoring capabilities and assist nursing staff with real-time user monitoring through camera configurations (Zhang et al., 2006). Notable examples include the intelligent nursing bed developed by Metrocare based on ergonomic principles and motion planning principles (Xin and Zhang, 2010), the integrated nursing bed robot with bed and wheelchair switching function developed by Devicelink Company (Luo, 2005), and the Gakusho series rehabilitation nursing bed robot developed by Matsushita Electric Company and Paramount Bed Company in Japan (Shi, 2014). In recent years, China has made significant progress in the field of rehabilitation nursing bed robots. Li Zhenqing et al. has developed a multifunctional nursing robot bed capable of addressing the high-efficiency nursing needs of long-term bedridden patients, including rehabilitation training, sitting, turning, eating, reading, writing, excretion, and entertainment (Li et al., 2012). Shanghai University of Technology has also developed a nursing bed robot that assists elderly individuals with back support, leg bending, turning over, and excreting. The system provides two nursing modes: active control and passive control. Furthermore, Zhang Hua et al. have invented a nursing bed integrated with a bathroom system, serving as an intelligent transportation tool while meeting the various daily care demands of families and hospitals (Zhang et al., 2011).

Intelligent assistive systems that facilitate proper standing and walking training for elderly with declining lower limb function have significant implications for the recovery of lower limb motor function and overall physical health. The renowned PAMM (Personal Aid for Mobility and Monitoring) system, developed by the Massachusetts Institute of Technology, consists of an intelligent walking machine and a smart walking stick device. The system utilizes force sensors as the primary input interface to drive the active wheel at the base of the system, which provides walking assistance to users (Dubowsky et al., 2000). To address the inconvenience of wearing devices, Honda has designed an intelligent lower limb assist system. This mechanical device, worn on the hip, assists elderly individuals with insufficient muscle strength by improving walking speed, increasing walking distance, enhancing gait uniformity, and monitoring the user’s heartbeat to adjust walking speed (Kusuda, 2009) automatically. Shenyang University of Technology and Kochi University of Technology in Japan have collaborated to develop a walking rehabilitation training robot for home care scenarios. This robot offers active training, passive training, and active-passive hybrid training modes. Equipped with an integrated omnidirectional mobile platform, it can operate within small indoor spaces without requiring a turning radius. The robot incorporates various interactive modes such as touch screen interaction, automatic navigation, and control based on the user’s walking direction and speed intentions, considering the exercise habits of frail elderly (Zhao et al., 2017). Compared to the steps involved in repeatedly placing and calibrating electrodes during rehabilitation training using a multi-channel EMG acquisition system, Zhao Xingang proposed a hand motion recognition system based on single-channel EMG signal decomposition. This system significantly improves motion recognition accuracy by 10% while enhancing operation convenience and user comfort. Its applications extend to daily sign language recognition, prosthetic hand control, and hand rehabilitation robot control (Zhao X. et al., 2019). Furthermore, Bai Jing from Southeast University proposed an autonomous home rehabilitation training and evaluation system based on workspace measurement. This system provides three-dimensional spatial image information, two-dimensional image information, and quantitative data to evaluate training outcomes, providing specific and easily understandable visual feedback for patients. The system overcomes the limitations of conventional treatment methods, enabling users to independently complete home rehabilitation training and evaluation without the supervision of a doctor (Bai et al., 2018). Various studies on indoor rehabilitation robots in elderly care scenarios have also been conducted by the Institute of Automation of the Chinese Academy of Sciences, Beijing University of Technology, Harbin Institute of Technology, and Huazhong University of Science and Technology.

#### 2.2.3 Multifunctional nursing robots based on human-robot interaction

**Figure 3:**
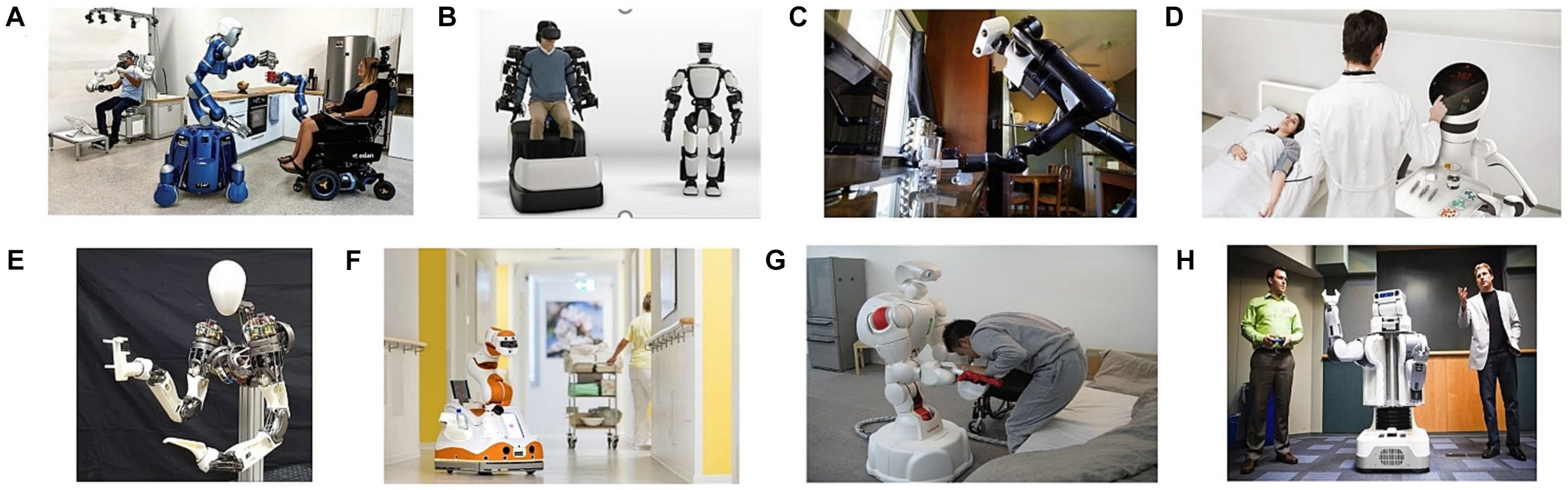
Multifunctional nursing robots based on human-robot interaction with a humanoid structure. **(A)** EDAN and Justin. **(B)** T-HR3. **(C)** Busboy. **(D)** Care-O-bot 4. **(E)** LIMS2-AMBIDEX. **(F)** Lio. **(G)** TWENDY-ONE. **(H)** PR2.

Multifunctional nursing robots based on human-robot interaction play a significant role in assisting nursing staff by effectively improving nursing ability and efficiency. The German space agency has developed the home care robot ecosystem SMILE (Vogel et al., 2021), which consists of the wheelchair robot EDAN, the humanoid robot Justin, and the tactile remote control device HUG (Figure 3A). EDAN integrates a force-controlled light manipulator with a five-finger intelligent hand, controllable via joystick, EMG signal, or EEG signal, to assist individuals with motor dysfunction. Justin is a robot with a wheeled chassis and a humanoid upper body equipped with a four-finger gripper. The humanoid upper body features two arms capable of executing various tasks, including making coffee, mopping tables, rinsing cups, and sweeping floors. The tactile remote control device HUG enables the remote control operation of EDAN and Justin. The system offers various operating modes with different levels of autonomy. TRI (Toyota Research Institute) has developed the T-HR3 (Figure 3B), a humanoid robot capable of safely supporting human activities at home or in medical institutions (Toyota Research Institute, 2021). Additionally, TRI has introduced the Busboy (Figure 3C), a new generation of home care robots that can perform relatively complex humanoid tasks. Busboy learns various household activities through human teachings, such as grasping objects and opening/closing doors, instead of being directly programmed for fixed tasks. Notably, the robot effectively links observed transactions with previous learning actions, enabling adaptive autonomous actions when encountering specific objects or scenes, even with slight task variations. To address the care needs of lonely, frail elderly, a Robotic home assistant, “care-o-bot,” has been developed (Hans et al., 2002). This series of robots not only performs a range of housework tasks but also provides emotional comfort through various interactive methods. The latest generation, Care-O-bot 4 (Figure 3D), supports a modular design, allowing users to configure it according to their preferences, resulting in improved quality and efficiency of care (Kittmann et al., 2015). The dual-arm robot LIMS2-AMBIDEX, based on tendon-driven technology, has been developed by KoreaTech in South Korea (Kim et al., 2018; Figure 3E). Each arm features seven degrees of freedom, and to reduce the inertia of the end effector, the corresponding drivers are positioned on the shoulder joint, utilizing a line drive transmission scheme. The design achieves an extremely lightweight structure of 2.63 kg below the shoulder joint, ensuring the safety and high-speed precision of dual-arm operations.

**Figure 4:**
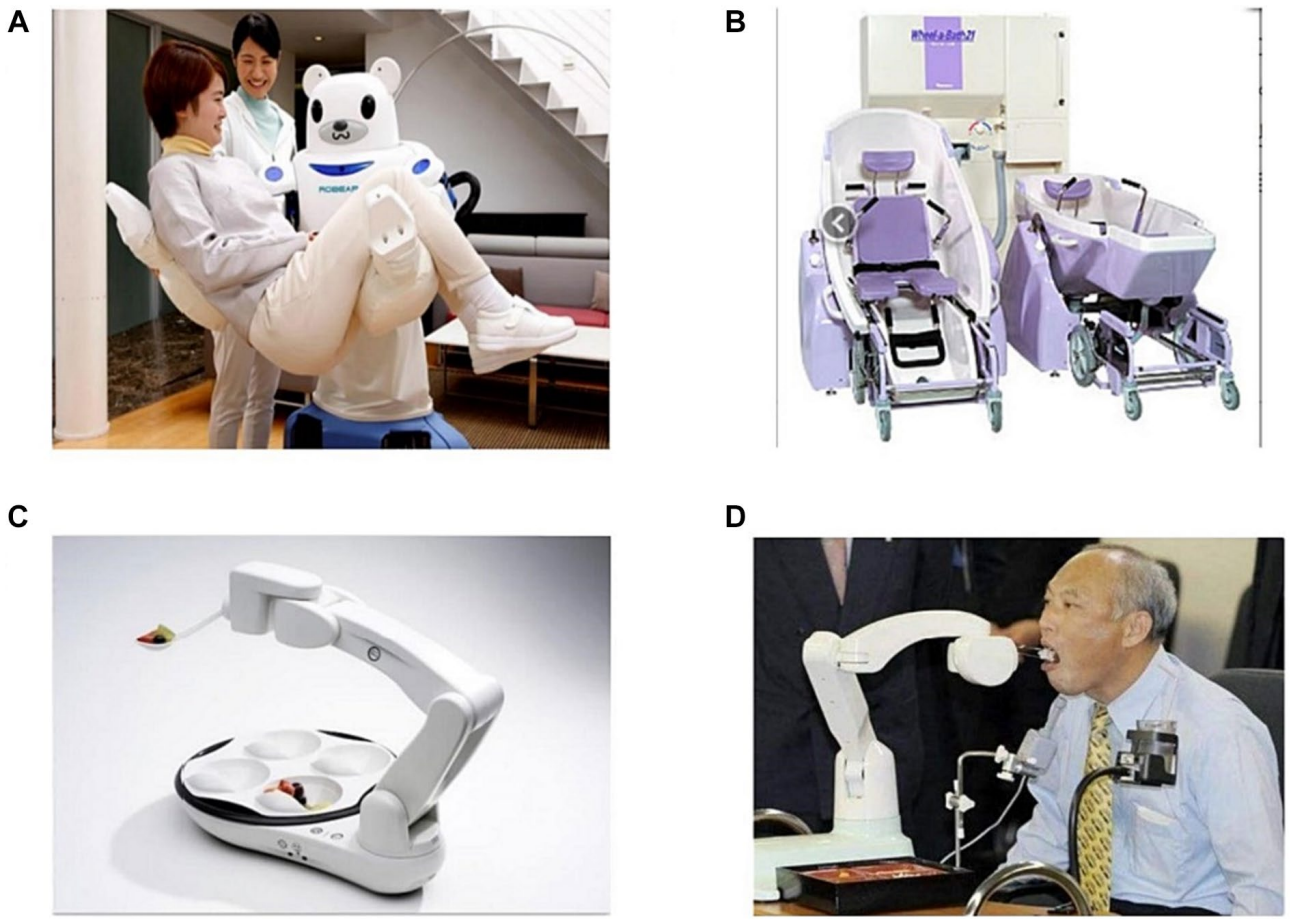
Multifunctional nursing robots based on human-robot interaction for special behaviors. **(A)** Robear. **(B)** Bishamon. **(C)** Obi. **(D)** My Spoon.

The Swiss F&P Robot Company has developed a multifunctional artificial intelligence (AI) nursing robot called Lio (Miseikis et al., 2020; Figure 3F), which integrates various capabilities such as walking, grasping, cognitive nursing, emotional support, disability assistance, rehabilitation therapy, patrolling, and delivery of living items. Lio has been successfully deployed in numerous nursing homes in Germany and Switzerland, effectively assisting nursing staff in providing targeted services. Lio is a nursing robot that efficiently assists the nursing staff in providing targeted services and is ideally suited for nursing homes, hospitals, nursing centers, and private residences. In 2007, Waseda University in Japan introduced a mobile-based multifunctional nursing robot named “TWENDY-ONE” (Hargrove et al., 2007; Vallery et al., 2008; Negro et al., 2016; Figure 3G). With 13 sensors on its hand, including a six-axis force sensor on its fingertips and distributed pressure sensors on its palm surface, TWENDY-ONE can perform delicate movements and provide a more human-like touch sensation through soft skin on its palm surface. Its arm is equipped with a high-power output driver that combines high-power.

output with a mechanical impedance mechanism, enabling it to perform precise grabbing and lifting tasks for patients. The Georgia Institute of Technology has developed the PR2 (Personal Robot 2) life behavior assistance robot (Rusu et al., 2009; Figure 3H). This system features two 7-degree-of-freedom anthropomorphic arms with a wrist equipped with a 6-axis force/torque sensor. The PR2 robot can autonomously assist bedridden users in body wiping tasks. The system includes an operator selection interface that allows autonomous selection of areas that require cleaning, including the upper arm, forearm, thigh, and calf (King et al., 2010). In the process of nursing, the repeated and repeated transfer services of the frail elderly bring excessive physical burden to the nursing staff, resulting in the majority of nursing staff in nursing homes suffering from chronic lumbar diseases. At the same time, most of the transfer assistive devices represented by traditional lifts are inconvenient to use, time-consuming and laborious and have low penetration rates. To this end, the RIBA series of robots have been developed to realize the safe and convenient transfer of lower limb disabled elderly in their living space. The third-generation Robear robots (Tak et al., 2010; Figure 4A) facilitate the safe and convenient transfer of lower limb disabled elderly within their living spaces. By analyzing posture, force, speed, acceleration, comfort, and other physical data, these robots can predict potentially dangerous behavior during the movement of the frail elderly. The robots are equipped with joint torque sensors and intelligent electronic rubber skin on their arms and chest, enabling them to accurately sense contact forces below 1 N and better understand the intentions of elderly individuals during mobile operations, ensuring patient health and safety (RIBA-II, 2021). The University of Pittsburgh has developed an assisted nursing robot that provides comfortable mobile assistance to users (Fan et al., 2018). Clinical experiments have demonstrated that this robot effectively reduces physiological and psychological pressure on nursing staff, thereby reducing overall management costs. Guo Shijie of Hebei University of Technology has developed a humanoid back-hugging mobile nursing robot with a three-degree-of-freedom chest mechanism to support the user’s chest during transfers (Guo et al., 2019). By imitating human back movements, this robot enables safe and efficient mobility and toileting for the frail elderly. The study integrates factors affecting mobility comfort into the human-robot system model, optimizing robot mechanisms and motion trajectories to effectively address the challenges faced by elderly individuals during mobility. Harbin Institute of Technology has determined the configuration and performance indicators of large-load manipulators based on the requirements of mobile tasks (Wu, 2020). They have successfully developed a two-degree-of-freedom joint system.

using multi-stage deceleration techniques, accompanied by the design of a compact six-degree-of-freedom manipulator. Combined with impedance control methods, these manipulators can lift paralyzed elderly individuals out of hospital beds. The robotic arm also features tactile perception and proximity sensing capabilities on its surface, ensuring safe and reliable nursing tasks through the proposed active and passive safety control methods. Zhejiang University and Yanshan University have developed a variety of transfer nursing robots that assist bedridden elderly individuals and people with lower limb disabilities in leaving hospital beds and moving conveniently. These robots include functions such as automatic folding, all-around automatic walking, automatic lifting, automatic toilet opening, and width adjustment. Furthermore, Jiang Yinlai and Zhao Donghui have invented a walking support robot with auxiliary standing and walking functions, effectively achieving safe and flexible standing assistance based on ergonomic principles in the standing process and intelligent wheelchair robot integration. Additionally, they have developed a multi-channel close-range sensor and a flexible control method based on human motion gait (Zhao, 2020). Significant research efforts have been made worldwide to enhance the mobility of elderly and disabled individuals through intelligent wheelchairs and their multimodal interaction technologies. Notable examples include the Wheelesley autonomous wheelchair robot (Yanco, 1998), the TAO Aicle intelligent wheelchair from Japan (Matsumoto et al., 2006), the Connie intelligent company KSI intelligent elderly function electric wheelchair, the RoboChair intelligent wheelchair developed by the Institute of Automation of the Chinese Academy of Sciences, the “Jiaolong” intelligent wheelchair developed by Shanghai Jiaotong University (Zhang, 2015), and the multimodal control wheelchair developed by Chongqing University of Posts and Telecommunications (Luo et al., 2012). These intelligent wheelchairs offer advanced features, such as multimodal human-robot interaction, efficient path planning, dynamic obstacle avoidance, and autonomous navigation, which greatly improve the mobility of elderly and disabled individuals. In Japan, the Bishamon care wheelchair bathing robot (He et al., 2019; Figure 4B) integrates an adjustable bathtub and an upper and lower separable wheelchair, significantly enhancing bathing efficiency and nursing care. Henan University of Science and Technology has developed a nursing robot bathroom system (Cheng, 2015), which includes bathing mechanisms and a robot that integrates auxiliary standing, friction, and shampoo mechanisms. This system assists users in standing and provides efficient cleaning services, ensuring a comfortable bathing experience.

Li et al. have developed a nursing shampoo robot that effectively addresses the safety control problem associated with existing shampoo robots while ensuring efficient cleaning of the user’s home (Li et al., 2017). In the domain of feeding assistance for frail elderly, notable robots include the Obi robot that helps disabled individuals eat unassisted by Design Robot Company in the United States (Automation, 2021; Figure 4C), the My Spoon meal-assistance robot by SECOM Company in Japan (Ishii, 2003; Figure 4D), and the intelligent feeding assistive robot developed by the University of Shanghai for Science and Technology (Xu et al., 2020). These innovative solutions provide effective auxiliary feeding schemes for frail elderly. Furthermore, Google’s Liftware Level anti-shake spoon has demonstrated its benefits for Parkinson’s patients by offering a stable grip and improving the eating experience (Liftware, 2021). Additionally, Krati has addressed the assistive dressing needs of frail elderly individuals s through the development of a deep learning-based framework. This framework accurately predicts clothing categories and identifies spatial coordinates for grip points, thereby streamlining the clothing operation process. Promising results have been achieved in fabric type detection and precise grasping point determination.

### 2.3 Companion robots based on emotional interaction

Companion robots based on emotional interaction have social and companionship capabilities, effectively assisting elderly individuals in addressing psychological and emotional problems such as isolation, emotion, stress, and loneliness, thereby improving their well-being. With continuous innovations in human-robot interaction (Goodrich and Schultz, 2007), behavior detection (Mohebbi, 2020), and autonomous navigation technologies (Marder-Eppstein et al., 2010), increasingly advanced socially interactive robots have emerged (Fong et al., 2003; Breazeal, 2004).

**Figure 5:**
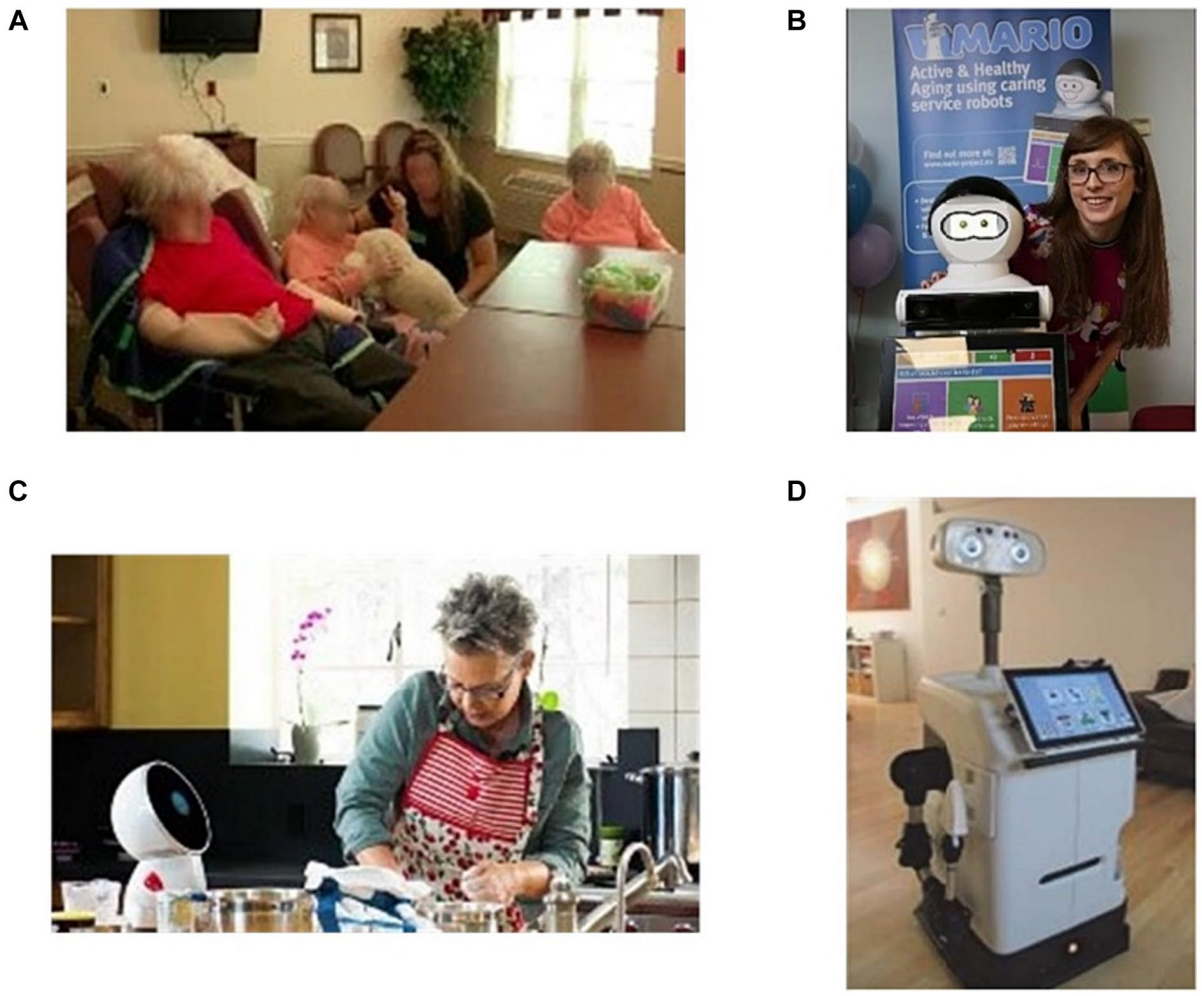
Companion robots based on emotional interaction. **(A)** PARO. **(B)** MARIO. **(C)** Jibo. **(D)** HOBBIT.

Within the scope of Horizon 2020, the European Union’s research and innovation framework program, numerous projects focused on elderly nursing services, including ENRICHME, SOCRATES, RADIO, and STREAMS, have been approved. Research indicates that older users are more receptive to robots that resemble pets or babies in both appearance and behavior (Breazeal and Scassellati, 2000). Consequently, various companion robots with pet-like or baby-like appearances have been developed to engage with frail elderly individuals daily. Notably, PARO, a seal-shaped robot (Figure 5A), incorporates tactile feedback, voice interaction, and posture perception to elicit positive emotions in users through real-time responsive behaviors such as head and flipper movements (Sabanovic et al., 2013; Hung et al., 2019; Chen et al., 2022). PARO has found utility in several nursing homes. NeCoRo robots, designed to resemble cats, are capable of expressing emotions like satisfaction, surprise, and happiness while responding appropriately to user emotions and actions (Nakashima et al., 2010). Clinical trials conducted in nursing homes show that NeCoRo effectively assists residents in improving communication and comfort among elderly individuals. AIBO, another companion robot, has been used in several clinical studies on the acceptability of geriatric care (Fujita, 2004). Participants exhibited a high degree of dependence on AIBO and reported reduced feelings of loneliness. Clinical experiments have further revealed that robot-assisted activities can help alleviate loneliness and enhance the activity and emotional state of patients with Alzheimer’s disease. MARIO (Figure 5B) alleviates the challenges of loneliness and dementia in elderly individuals through the development of companion robots (MARIO, 2021). A clinical study of MARIO has revealed various factors affecting its acceptability, including facial appearance, knowledge of user preferences, practicality, and the ability to connect users with their family and friends (Casey et al., 2016). The Jibo social robot (Figure 5C) conducted a three-week “facilitating social interaction experiment” on 19 participants in an assisted living community in California (Jibo, 2023). By utilizing natural language processing techniques to provide information pertaining to daily activities obtained from the internet, Jibo enabled remote nursing staff to establish a sense of telepresence within the user’s home (Roy et al., 2000). Many nursing studies have highlighted the significant influence of emotional, behavioral, and environmental factors on the nursing experience of elderly individuals (Hirsch et al., 2000). In conclusion, social robots have demonstrated their effectiveness in enhancing user engagement and fostering social connections. Nevertheless, these advancements also shed light on users’ concerns regarding security, privacy, and the collective apprehensions surrounding social robotics.

Based on completing the accompanying behavior, the robot can provide extensible behaviors such as feeding reminders, abnormal behavior recognition, and entertainment activities.

Mabu, a personal healthcare companion robot, was introduced in 2015 ((My Website, 2023)). This robot engages in social interactions with elderly individuals and creates personalized conversations for each user based on classical psychological models of behavior, helping to address issues related to chronic diseases, such as medication intake. Stevie, developed by Trinity College Dublin (McGinn et al., 2020), interacts with elderly individuals through a combination of sensory data, including gestures, speech, and facial expressions, with high acceptability. Mabu and Stevie robots are primarily employed in reminder scenarios and do not include behavior monitoring or data collection and analysis systems. The HOBBIT project, funded by the European Union, aims to develop a socially assistive robot (Figure 5D) capable of tasks such as picking up and carrying, emergency recognition, fitness planning, and reminders while ensuring the safety of elderly individuals (Bajones et al., 2018). The project introduces.

the concept of interactive care, enabling the robot to learn the user’s habits and preferences to adapt its behavior. ZORA presents two commercial robot solutions (Zorarobotic, 2021). ZoraRobot, based on the Nano platform, integrates healthcare applications that assist elderly individuals in interactive therapy and entertainment activities and have been introduced in nursing homes and retirement communities in the United States. Zorabot, on the other hand, is based on the Pepper robot platform and incorporates medical applications, providing functions such as visiting, greeting and returning, inquiries, and medical assistance. Blue Frog has developed a robot named BUDDY (BUDDY, 2021), which enhances care for elderly individuals by providing companionship and assistance in their daily activities. For instance, it can remind them to take medication, make appointments, deliver items, or detect abnormal behaviors such as falls. In the European Union’s 7th Research Framework Programme (FP7), several elderly care projects combine robotics technology with ambient intelligence to achieve panoramic semantic integration for assisted home care, facilitating independent living for elderly individuals (Saunders et al., 2013). The CompanionAble project effectively addresses the social integration and home care challenges faced by elderly individuals with chronic cognitive impairment in Europe (Badii et al., 2009). The GiraffPlus project combines sensor networks to provide a remote-controlled robot for monitoring activities within the home environment. The project particularly emphasizes the emotional aspect of user interaction to meet their needs and capabilities (Coradeschi et al., 2014). The Mobiserv system comprises a socially interactive robot, wearable smart clothing, and a smart home environment, aiming to support the daily lives of elderly individuals with comprehensive care services focused on health, nutrition, happiness, and safety, thereby improving their quality of life and care efficiency (Nani et al., 2010; Esposito et al., 2014).

Regarding cognitive assistance, LIZA (Le and Wartschinski, 2018) has emerged as a widely adopted cognitive companion that enhances older adults’ reasoning and decision-making abilities through continuous human-robot interaction. This system employs natural language interaction based on 66 topic texts to impart knowledge to elderly individuals about one or more subjects. By posing synthesized questions and adapting to the individual needs of elderly individuals during each interaction, LIZA autonomously and subconsciously corrects its guidance criteria. Furthermore, social assistance robots (Andriella et al., 2018) have also found extensive applications in cognitive assistance. Through efficient interactions, these robots encourage elderly individuals to grasp specific tasks or engage in cognitive exercises such as the Syndrom-Kurztest neuropsychological battery (SKT). They exhibit adaptability to the diverse responses of different older adults and provide support and assistance at multiple levels of interaction.

In summary, current companion robot technologies are predominantly focused on specific domains. In comprehensive elderly care scenarios, multiple systems must collaborate to provide comprehensive support, which poses challenges in effectively managing these systems and accurately monitoring the health conditions of elderly individuals. Moreover, using visual sensors in home care scenarios raises privacy concerns. Therefore, it is necessary to establish appropriate regulations to address the ethical and technological boundaries, aiming to minimize threats to the privacy of elderly individuals. Furthermore, the issue of acceptance by the elderly population presents a significant challenge in the widespread adoption of robotics in elderly care. The difficulty in operating high-tech products and the gap between social experiences and expectations contribute to the resistance among some elderly individuals to utilize such technologies. Research shows (Sixsmith, 2013) that the acceptance of robots depends not only on the personalized functionalities they offer, such as entertainment, status enhancement, and tangible benefits, but also on the inherent characteristics of individuals, including age, needs, gender, technological experience, cognitive abilities, education, culture, roles, expectations, attitudes toward robots, and the inherent characteristics of robots, such as safety, usability, intelligence, appearance, human likeness, facial expressions, physical size, gender, personality, and adaptability (Young et al., 2009; Frennert et al., 2012). Therefore, effectively handling the practicality and social aspects while satisfying the essential performance requirements and catering to the individual differences of users is crucial to the successful deployment of companion robot technologies in elderly care scenarios and to enhance acceptance among the elderly population.

## 3 Safe interaction methods

The safe human-robot interaction method is one of the most critical research areas in elderly care scenarios. This technology encompasses collision protection between users and robots within shared spaces while comprehensively considering various ways in which harm can be inflicted upon frail elderly individuals, from physical contact to psychological effects. This paper comprehensively describes four aspects of safe interaction methods: control methods, motion planning, behavior prediction, and psychological factors.

### 3.1 Control-based safe interaction methods

Control strategies are commonly used to achieve safe human-robot interaction, without the need for complex predictive models or planning strategies, while still demonstrating high system robustness. Among these, safety improvements in human-robot interaction can be split into two distinct phases: pre-collision safety and post-collision safety.

Pre-collision safety control methods are measures taken before an actual collision between humans and robots occurs. These methods can be achieved by ensuring collision avoidance as a primary objective or by constraining critical parameters of the robot. For instance, collision prevention can be achieved by limiting the robot’s speed or energy, using defined safety zones, maintaining tracking separation distances, guiding the robot away from humans, and other methods. The common approach involves controlling safe human-robot interaction by setting thresholds for key system parameters, such as joint velocity, energy, or force exerted by the robot. One method combines a force threshold-based foot motion control strategy with an improved robot body center of mass motion for enhanced performance when walking on uneven terrain (Heinzmann and Zelinsky, 2003). Another approach by Broquere et al. utilizes piecewise cubic polynomial chains to construct trajectory chains that limit jerk, acceleration, and velocity for pre-collision safety control (Broquere et al., 2008). While these methods may be overly conservative without imminent collision threats, they provide pre-collision safety by globally constraining the robot’s motion without relying on accurate and robust detection and tracking of human positions. Another method of pre-collision control involves decelerating or stopping the robot by utilizing safety zones or separation distances to prevent collisions. This method uses depth sensors to estimate the distance between the robot and static or moving obstacles. It combines real-time distance measurements with estimated obstacle velocities to adopt a repulsive vector-based collision avoidance controller (Buizza Avanzini et al., 2014). De Luca et al. also propose a method utilizing a distributed distance sensor and optimization of robot body sensor node positions for safety (Flacco et al., 2012). Compared to threshold-based methods, this method offers greater flexibility but also requires robust and low-latency tracking of humans within a given space. Incorporating risk criteria and field computation into control algorithms can prevent robot collisions. For instance, potential field methods define repulsive vector fields that guide robot motion, adjusting the trajectory based on changing dynamic environmental factors to ensure safety during complex behaviors (Khatib, 1986; Kovács et al., 2016). This method allows for more intricate collision avoidance beyond adjusting the robot’s speed. However, the method’s effectiveness depends on the strategy employed in constructing the potential field. In elderly care scenarios, customizing strategies involve factors beyond separation distances, such as approach direction, emotional states, and human gaze direction. Therefore, in the case of unexpected or unpredictable contact, post-collision control methods minimize harm by switching to different control strategies, differentiating between intentional and unintentional contact, and allowing safe physical contact when necessary to achieve effective collaboration. The first step in implementing post-collision control methods for safe human-robot interaction is to detect whether a collision has occurred. Sharkawy et al. propose a collision detection method based on a multi-layer feed-forward neural network, utilizing robot position and joint torque sensors for detecting collisions between operators and robots, applicable to any robot equipped with joint torque sensors (Sharkawy et al., 2020). Geravand et al. present another method using motor current measurements for detection and response, eliminating the need for torque sensors (Geravand et al., 2013). This system is designed for robots with closed-loop structures, capable of distinguishing between intentional collisions and switching the robot into a collaboration modality upon collision detection. Golz et al. propose a method utilizing machine learning and physical contact models to distinguish between intentional and unintentional robot collision detection, employing non-linear support vector machines for classification by observing real collision data and extracting a set of features. As interaction scenarios become more complex, effectively distinguishing between intentional and unintentional contact in collision detection systems becomes critical to ensuring safe interactions. When intentional contact is recognized, the robot must accurately infer the user’s collaborative intent and provide optimal support actions during the interaction rather than simply minimizing damage by avoiding collisions or switching control. Luca and Flacco proposed an inference framework based on user gestures and speech to determine whether users intend to enter a collaboration modality and specify body-specific parts where robot contact is allowed or prohibited, such as the user’s head (De Luca and Flacco, 2012). Additionally, these frameworks can effectively estimate interaction forces at contact points and control the robot to ensure that they do not exceed predetermined thresholds. In elderly care scenarios, collaborative contact requires specific consideration of the actual contact points and monitoring and limiting the effects of forces during an interaction.

### 3.2 Motion planning-based safe interaction methods

Motion planning-based safe interaction methods employ robot path planning and motion planning to avoid collisions and ensure safety. In the process of motion planning, human-related constraints, such as separation distances and human gaze directions, are directly incorporated into the motion planner’s cost functions and objective functions to actively avoid collisions and generate comfortable and user-acceptable motions, thereby achieving optimal motion planning strategies in closed-loop scenarios. Motion planning takes a more proactive approach than control-based methods to ensure interaction safety. Research has shown that, in some instances, traditional control methods for collision avoidance may result in lower safety and efficiency compared to perception-based motion planning methods and may negatively impact the user’s psychological safety (Lasota et al., 2014). Furthermore, motion planning algorithms incorporating safety operators apply to almost any robotic platform, such as manipulator manipulation and robotic navigation, demonstrating good functional generality and portability. These algorithms primarily include planners based on human closure constraints and motion planners based on geometric and task constraints. Compared to threshold-based control methods, the parameters of motion planners can minimize risk operators throughout the motion process rather than just approaching safety thresholds. Several motion planning algorithms have been developed that take human constraints into account. For instance, for scenarios involving human-carrying mobile platforms, Morales et al. developed the HCoPP (Human Comfort Path Planner), which takes into account user preferences, such as the desired distance from walls when walking in corridors and visibility at corners while approaching turns, to design driving paths that provide a comfortable and pleasant experience for the user (Morales et al., 2015). In the spatial zones of human-robot collaboration, due to the inherent uncertainty in user behavior, motion planning strategies need to consider the ability for rapid re-planning based on geometric and task constraints. Interaction safety zones and buffer zones can be encoded using geometric constraints, while additional information guiding the robot’s motion can be modeled using task constraints, such as leveraging previous observations to predict the occurrence of future events. By hierarchically encoding task constraints and geometric constraints and combining them, complex search spaces can be efficiently traversed, effectively eliminating illogical solutions and local optima, thereby accelerating computation speed Combined Task and Motion Planning for Mobile Manipulation (Wolfe et al., 2021).

### 3.3 Perception and prediction-based safe interaction methods

Accurate perception and prediction of each other’s behaviors and actions are crucial to ensure safe interaction between human users and robots in scenarios involving mutual proximity, contact, and dynamic interaction. This capability needs to be extended to all members of multi-robot systems involved in assistive behaviors alongside users. Perception of human activity states encompasses the perception and prediction of actions. In pursuit of this goal, research focuses primarily on behavior analysis by analyzing the underlying features of motion signal sensors, physiological signal sensors, visual sensors, and depth sensors.

In the field of elderly care, robots need to monitor and discriminate the behavior of elderly individuals in indoor environments, such as homes, which is crucial for enhancing safe interaction. These behaviors include behavior monitoring, fall detection, abnormal behavior detection, and medication monitoring, which significantly improve the safety of assisted living in homes. For instance, in designing applications for the frail elderly, an effective and common method for fall detection involves utilizing the three-dimensional depth data from depth cameras to determine the distance from the human centroid to the ground and to analyze the vibration characteristics. However, the robustness of traditional global visual sensors is compromised when there are occlusions between users and robots. A fall recognition algorithm based on a set-source filter can effectively identify various abnormal gaits, including falls and dragging gaits (Zhao D. et al., 2019). Activity prediction using image data has been explored by Azorin-Lopez et al., where they establish normalized activity vectors to describe RGB (Red Green, and Blue) interaction data, eliminating dependence on time or event sequences (Azorin-Lopez et al., 2014). Shah et al. utilize a time-series analysis method, defining multivariate Gaussian distributions for each motion time step based on tracked human arm degrees of freedom (Perez-D’Arpino and Shah, 2015). The system utilizes the learned model for Bayesian classification of the initial phase of motion to predict the direction of a person’s movement and select the robot’s action with minimal interference. Intention recognition is a prerequisite for achieving safe human-robot interaction, particularly for frail elderly. Intention recognition can be accomplished through explicit and implicit methods. Explicit methods directly convey the robot’s intent and planned behavior through language, visual cues, auditory signals, and other explicit means. In cases where explicit expression of motion goals is not desired, the implicit method implicitly embeds subtle cues through action performances to convey motion intentions. By combining these methods in specific elderly care scenarios, human-robot behavior becomes more predictable, allowing for more effective motion planning and ensuring safe interaction. Intention recognition needs to be specifically defined in conjunction with particular tasks and can involve the recognition of individual action switches, transitions between multiple discrete actions, and continuous information. These methods have been applied in research on facial recognition-based user emotion detection, language recognition, action intention recognition, and cognitive emotion regulation methods, providing a solid research foundation for human-robot interaction technology in the context of elderly care scenarios (Li, 2013; Liu, 2015; Liu et al., 2016; Lu, 2017). Particularly in the domain of action intention recognition, various applications include control of prosthetic grasping, dexterous manipulation of prosthetics, control of brain-computer interfaces, and assistance in standing and walking. These motion signal perception-based studies mainly utilize position sensors, angle sensors, and force sensors to capture the motion state of the user’s limb joints, which are then fed back to the control unit to form a closed-loop control system. This approach enables smooth control, significantly improving precision and accuracy and preventing secondary injuries. For instance, Xu Wenxia et al. propose a multi-sensor fusion-based motion control method for assistive walking robots that can adapt to users’ walking intentions, predict potential falls during usage, and take corresponding fall prevention strategies. The effectiveness of this method is demonstrated through experiments (Xu et al., 2016). Another technique for human action prediction does not directly infer sensor data but represents discrete actions or task steps in a labeled form. Task models are used to infer actions taken and combined with sequence matching and probability recognition methods for prediction. In this regard, the objective of perception systems is action detection. Nikolaidis et al. encode human-robot collaborative tasks based on Markov decision processes and accurately predict the outcome of human actions (Nikolaidis and Shah, 2013). They subsequently propose a hybrid observable Markov decision process (Nikolaidis et al., 2015). In this work, the system clusters action sequences, learns reward functions for each cluster through inverse reinforcement learning from joint action demonstrations, and automatically learns user models. The robot then utilizes these models to predict user types for anticipating user behavior and executing appropriate expected behaviors.

In order to create a safe, comfortable, and natural assistive environment or rehabilitation training system for elderly individuals, research on perception and prediction between robots and elderly individuals play a crucial role. This research not only identifies the current states of elderly individuals and robots but also facilitates interaction between them. It can construct a closed-loop control system, improve the control accuracy of the system, and use the information of force and position to realize the compliant control of the robot. In addition, the research of perception and prediction can also be used as an evaluation signal to participate in the evaluation and calibration of rehabilitation robot performance (Lloyd and Besier, 2003; Balbinot and Favieiro, 2013). Accurate perception combined with high-quality control methods can create a safe and comfortable care and rehabilitation environment for elderly individuals, significantly improving nursing safety and nursing efficiency.

### 3.4 Psychological factor analysis based safe interaction methods

Enhancing psychological safety is a key factor in users’ acceptance of elderly-care robots. Maintaining psychological safety means ensuring that users subjectively perceive their interactions with the robot as safe without causing any psychological discomfort or stress, whether due to the robot’s actions, appearance, speech, posture, social behavior, or other attributes. Previous research has shown that relying solely on physical safety measures is insufficient to improve users’ sense of safety and comfort (Lasota and Shah, 2015). Therefore, in human-robot interaction, a crucial method for ensuring psychological safety is appropriately adjusting the robot’s behavior, which can be categorized into adjustments based on robot characteristics and social factors characteristics. Researchers commonly evaluate the effectiveness of psychological safety through three evaluation methods: questionnaire surveys, physiological indicators, and behavioral indicators.

Research on adjustments based on robot features focuses primarily on psychological safety factors by modifying various parameters of robot motion, such as speed, acceleration, or the degree of proximity to human characteristics, to make human-robot interaction more comfortable. Previous studies have quantified user comfort during human-robot interaction by establishing human comfort models. For instance, dynamic comfort models have been developed to enhance the comfort of the interaction process by adjusting parameters such as robot speed and distance from the user (Wang et al., 2018). Sisbot and Alami found that physical safety alone is insufficient to achieve user-acceptable human-robot interaction and that any behavior that could cause fear or discomfort in humans must be avoided. Therefore, they developed a safety-compliant motion planner (Sisbot and Alami, 2012). Mainprice et al. generated reactive actions that satisfy human comfort by introducing interaction constraints in the motion planner (Mainprice et al., 2011). Additionally, it is necessary to consider research and adjustments related to robot features and understand the influence of social factors (Haring et al., 2013; Costanza et al., 2014; Obaid et al., 2016). Factors such as different robot postures, appearance design, and different types of background sounds can affect user comfort. Another method to enhance interaction safety through psychological factors is to adhere to social norms, including considering the influence of cultural and personality differences. It is essential to translate social habits observed in human interactions into methods for interacting with robots and to consider the impact of robots violating social conventions on users’ psychological safety. Researchers such as Joosse have found that users are highly sensitive to the extent to which robots respect social norms when invading personal space, and their reactions to robot invasions of personal space can be even more potent than those of humans (Joosse et al., 2013). A six-week clinical study by Walters assessed participants’ behavioral preferences when interacting with robots in a home environment. The results showed that even without safety hazards, robot failures directly increased the expected distance between humans and robots. Furthermore, studies have revealed that compared to the distances at which users willingly approach robots, they allow robots to approach them at greater distances within physically constrained areas. Ghazali et al. found that users tend to trust robots with human facial features more, and most users exhibit higher psychological reactions when interacting with opposite-sex robots (Ghazali et al., 2018). Furthermore, studies have indicated that users from different cultural backgrounds have varying standards for appropriate proximity when robots move toward a group of people, and user differences in traits, culture, and experiences can influence behavioral preferences (Joosse et al., 2014).

## 4 Future development directions of elderly-care robots

### 4.1 Multi-heterogeneous nursing robot system and their collaborative control technology

Due to the strong individual variations in physical conditions among frail elderly individuals, the development of rigid-flexible coupled robots for the care of frail elderly and their human-robot collaboration is gradually becoming a new trend in achieving universal high-quality elderly care. Rigid-flexible coupled robot systems, by seamlessly combining flexible actuation and transmission with rigid support and execution capabilities, can effectively simulate human nursing processes and compensate for the shortage of staff nursing. This provides a new safe, efficient, and accurate robotic nursing solution. To address the deficiencies in contextualization and functionality in daily life, there is a need to establish an overall system for a variety of heterogeneous elderly care service robots tailored to complex caregiving scenarios, alongside a robust communication system. Furthermore, it is crucial to develop task planning and collaborative control methods customized for specific application scenarios, assisting users in achieving safe and smooth state transitions to support continuous activities in daily life. Additionally, optimizing the structure and nursing modes of elderly-care robots based on the analysis of human motion functions and human factors engineering is an essential direction for the future development of nursing robots, ensuring a balance between safety and adaptability in assisting daily living.

### 4.2 Establishing a new model of smart home care for elderly individuals based on robotic nursing systems and sensor networks

Compared to health care, nutrition, social interaction, and other daily living support services provided by adult day care centers, long-term care facilities, and nursing homes, elderly individuals prefer to live in their own homes and enjoy the sense of familiarity, self-confidence, independence, and sense of accomplishment brought about by self-care activities. Therefore, establishing a home care model that meets specific user needs, combined with intelligent home sensor networks and robotic nursing technologies, can provide users with context-aware ubiquitous computing applications and home automation services. What’s more, this technology is a future development trend. The framework of home-based elderly care systems should possess adaptability to efficiently cater to the diverse needs of the elderly population with individual differences. It should correctly manage sensor network systems, elderly smart systems with medical and metric monitoring, life-assisted nursing robots, and companion robots based on emotional interaction. Additionally, standardized data communication methods and cloud management systems need to be established to realize a new model of smart home-based elderly care.

### 4.3 Future directions for the safety of interactive control and compliance of frail elderly in complex scenarios

Considering the physiological characteristics of frail elderly, such as decreased continuity, stability, and susceptibility to misleading signals in their diminished motor functions, rigid robot operation based solely on experience can easily lead to unnoticed secondary damage or even life-threatening hazards. The extraordinary physical conditions of frail elderly and their demands for nursing safety impose strict requirements on the safety and compliance of nursing robots. It is essential to combine foundational theories and key technologies from the fields of medicine, information, and mechanics to enhance the compliance and safety of robot operations. Simultaneously, for specific caregiving tasks, selecting effective intent recognition carrier signals for frail elderly individuals and effectively combining their respective advantages, establishing a method for synchronized acquisition of multi-source signals, feature extraction, uniform representation, and joint recognition is crucial. This method enhances the estimation accuracy and stability of human intent and target information, enabling efficient information exchange between frail elderly individuals and caregiving robots, and is a key component in the development of caregiving robots for frail elderly individuals. Moreover, designing efficient control techniques, establishing mappings between intent and interfaces, and incorporating collaborative task constraints while integrating elderly individuals as part of the closed-loop control system, allows robots to adapt to the intentions of elderly individuals, ensuring high fault tolerance and compliance in their responses. This addresses essential issues in nursing robots. To further enhance robots’ safety and caregiving capabilities, it is imperative to integrate the physiological characteristics of frail elderly individuals and break through the core technologies of human-robot coupling dynamics, service robot design, intention recognition for frail elderly, and human-robot coordinated control. This will establish a new principle and theoretical framework for rigid-flexible coupled robots tailored for frail elderly care.

### 4.4 Exploring the critical issues of robotic nursing technology, acceptability, and ethics

The development of robotic technologies for elderly nursing brings benefits to frail elderly but also raises concerns regarding privacy rights, interpersonal interaction, quality of care, acceptability, and ethical considerations between the elderly and nursing robots. Key ethical dilemmas include striking a balance between the need for supervision and privacy protection, the potential misguidance caused by one-sided emotional bonds with elderly individuals, restrictions on elderly individuals’ behaviors due to the autonomy of nursing robots, the insufficient acceptability resulting from the objectification risks in robot nursing services, and concerns about the deterioration of interpersonal skills in elderly individuals. Therefore, it is necessary to establish effective, ethical principles considering the differences between robot technology and human ethics while meeting the principles of specificity, flexibility, and safety to ensure that elderly-care robots can truly enter and benefit every household.

## 5 Conclusion

In summary, the application prospects of elderly-care robots in assisting the frail elderly in daily living assistance, nursing, and rehabilitation training are extensive, making it one of the most challenging research areas in robotics. Over the past 30 years, elderly-care robots have gradually transitioned from laboratory research to clinical applications, giving rise to a significant number of distinctive life-assisted nursing robots, companion robots based on emotional interaction, and intelligent robots based on medical functionality and physiological index monitoring. This progression has greatly advanced both the research and application levels in this field. Given that frail elderly individuals have direct physical contact with robots, the methods for safe human-robot interaction are crucial scientific issues affecting interaction performance. Utilizing motion control, motion planning, prediction methods, and psychological factors are essential means and core technologies for achieving safe human-robot interaction strategies. Future research will focus on the collaborative assistance of heterogeneous assistive robots, the establishment of home-based elderly care models based on robots and sensor networks, the optimization of robot flexibility design, and the improvement of robot acceptance. These efforts will ultimately enhance the level of service and nursing provided by elderly-care robots.

## Author contributions

DZ: Funding acquisition, Project administration, Supervision, Writing – review & editing, Conceptualization, Resources. XS: Writing – original draft, Investigation. BS: Data curation, Investigation, Methodology, Writing – review & editing. ZY: Formal analysis, Investigation, Validation, Writing – review & editing. JY: Conceptualization, Project administration, Supervision, Writing – review & editing. HL: Formal analysis, Supervision, Validation, Writing – review & editing. YJ: Writing – review & editing. YH: Funding acquisition, Project administration, Supervision, Writing – review & editing.
